# Toxicological Effects and Health Impacts of Per- and Polyfluoroalkyl Substances (PFAS) in Humans

**DOI:** 10.3390/toxics14050374

**Published:** 2026-04-26

**Authors:** Jie Ma, Ge Gao, Bitan Meng, Xinni Wei, Long Zhao, Zaiming Ge

**Affiliations:** Center for Environmental Health Risk Assessment and Research, Chinese Research Academy of Environmental Sciences, Beijing 100012, China; 13068009060@163.com (J.M.); gaoge_20002018@163.com (G.G.); bitandream@163.com (B.M.); 15141000600@163.com (X.W.)

**Keywords:** per- and polyfluoroalkyl substances (PFAS), exposure, toxicity, mechanism, health

## Abstract

Per- and polyfluoroalkyl substances (PFAS) are a class of synthetic chemicals notable for their high persistence and extensive applications. With the advancement of detection technologies in recent years, PFAS have been frequently identified in environmental media and human biological samples, raising significant global concerns about their potential health risks. PFAS exhibit distinctive toxicokinetic behaviors, including efficient absorption, strong protein binding, limited metabolism, and slow excretion, which lead to prolonged biological half-lives and considerable bioaccumulation in humans. These properties contribute to a range of adverse health outcomes, such as endocrine disruption, immune suppression, liver damage, reproductive toxicity, carcinogenic potential, and cardiovascular disease. This review synthesizes evidence on PFAS-associated health risks from a multisystem, multitarget perspective, elucidating the key molecular pathways involved, thereby providing a scientific basis for understanding their complex toxicological effects and for developing targeted prevention and control strategies. Future research should prioritize characterizing the toxicological profiles of individual PFAS compounds, evaluating the health impacts of combined (mixture) exposures, and assessing risks associated with chronic, low-dose exposure to support the development of public health strategies and regulatory decisions.

## 1. Introduction

Per- and polyfluoroalkyl substances (PFAS), a prominent class of synthetic compounds, have attracted widespread global attention due to their potential risks to human health and ecological systems. The European Chemicals Agency has estimated that approximately 4.4 million tons of PFAS could be released into the environment by 2053 without immediate action to restrict their use [1]. This potential massive release is attributed to the widespread application of these persistent chemicals in consumer products and industrial sectors since the 1940s [2]. Due to the exceptionally strong carbon-fluorine (C-F) bonds, these compounds exhibit extreme chemical and thermal stability, earning them the moniker ‘forever chemicals’ [3,4]. Consequently, PFAS are now pervasive environmental pollutants, detected in ecosystems from the remote Arctic to urban settings, indicating long-range transport and deposition [5]. Furthermore, PFAS possess unique amphiphilic properties, featuring both hydrophilic functional groups and hydrophobic fluorinated alkyl chains. They also have diverse monomeric structures, including linear, branched, and cyclic, as well as varying carbon chain lengths [6]. These specific structural characteristics not only significantly enhance their environmental persistence and bioaccumulation potential but also facilitate their penetration into biological membranes, leading to widespread distribution within the human body. This structural diversity results in highly variable toxicokinetics, which drastically complicates research on their specific toxicological targets and human health effects.

PFAS can enter the human body through multiple pathways, including drinking water, food, and the inhalation of contaminated air and dust [7]. To date, PFAS have been ubiquitously detected in human matrices such as blood, urine, breast milk, and specific tissue samples. While legacy PFAS are commonly detected in the general global population at concentrations ranging from a few to tens of nanograms per milliliter (ng/mL), levels in populations residing in contaminated areas, occupationally exposed groups, and within diseased tissues (e.g., liver tumors) can be orders of magnitude higher, reaching concentrations associated with significant health risks [8]. This ubiquitous human exposure represents a critical public health challenge within the broader context of environmental health. In fact, the World Health Organization estimates that environmental factors contribute to 24.3% of global premature deaths and 28.1% of deaths in children under five annually [9]. Within these environmental factors, chemical pollution, particularly from synthetic chemicals, is a major driver. It is estimated to contribute to approximately 1.8 million deaths annually on a global scale [10]. Given this substantial global health burden, the specific toxicological targets and health effects of widespread PFAS exposure require urgent elucidation. Recent epidemiological risk assessments indicate that current human exposure levels in many regions exceed newly established safety thresholds, posing substantial health risks to the general population. For instance, benchmark doses for immunotoxicity, derived from child cohort studies, are as low as 1.3 ng/mL for serum PFOS and 0.3 ng/mL for PFOA; biomonitoring data indicate that average serum concentrations in the general populations of numerous countries already greatly exceed these levels [11]. Furthermore, dietary exposure, particularly through fish consumption, often leads to exceeding strict intake guidelines. The European Food Safety Authority (EFSA) recently updated its tolerable weekly intake (TWI) to 4.4 ng/kg body weight per week for the sum of four major PFAS. However, median PFAS concentrations in freshwater fish from the U.S. Great Lakes and European waterways reach 13.8 and 22 μg/kg, respectively. These concentrations are orders of magnitude above the new EFSA threshold, demonstrating that even modest regular fish consumption can independently drive hazardous exposure levels [12]. A growing body of epidemiological and toxicological evidence demonstrates that PFAS exposure is closely associated with multi-organ adverse health outcomes, including endocrine disruption, immunotoxicity, liver toxicity, reproductive toxicity, increased risk of cardiovascular disease, and certain cancers (e.g., renal and testicular cancers) [13]. This is corroborated by specific incidents, such as the severe environmental contamination event in the Veneto region of Italy, where PFOA groundwater concentrations reached up to 20 μg/L, which was associated with increased risks of kidney cancer, diabetes, cerebrovascular diseases, and myocardial infarction in the exposed population [14]. Additionally, PFAS bioaccumulate in specific internal organs such as the liver, kidneys, and bones, driving localized toxicity like chronic kidney disease and liver dysfunction [15].

Current research is shifting from high-dose, single-substance studies to investigating complex, low-dose, long-term mixture exposures and elucidating underlying molecular mechanisms. However, because PFAS encompass thousands of homologues and isomers with vastly different toxic potencies, distinct effects on various organ systems, diverse susceptibilities across different populations, and highly complex underlying mechanisms, a comprehensive and systematic understanding of their overall toxicity and health effects remains limited [16,17].

Therefore, this study aims to provide a comprehensive review of human exposure, toxicological effects, and the underlying mechanisms of PFAS. The primary objectives of this article are to: (1) summarize the current state of human exposure to PFAS and their toxicokinetics; (2) systematically evaluate the epidemiological and toxicological evidence regarding their adverse health effects across multiple organ systems; and (3) elucidate the complex molecular mechanisms driving these health outcomes. Ultimately, this review aims to provide a robust scientific foundation for future health risk assessments, environmental management, and the development of effective public health policies regarding PFAS contamination.

## 2. Toxicokinetics of PFAS in the Human Body

**Figure 1:**
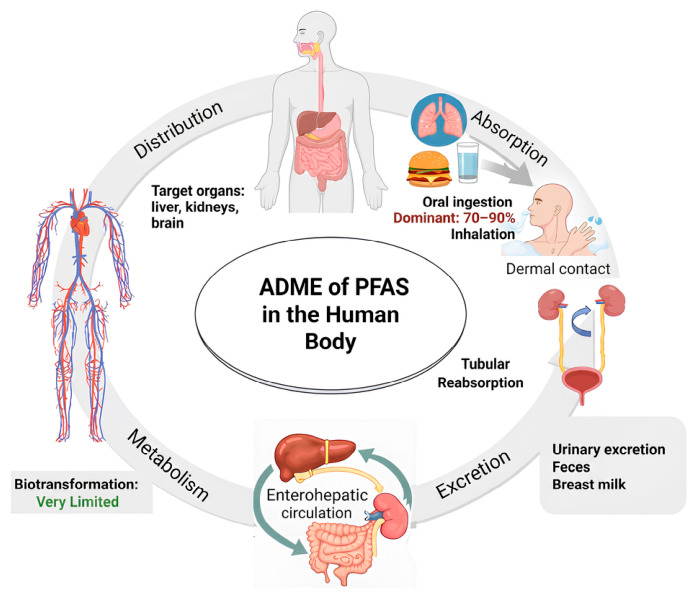
The primary absorption, distribution, metabolism, and excretion (ADME) of Per- and polyfluoroalkyl substances (PFAS) in the human body.

Compared to traditional lipophilic persistent organic pollutants (POPs), PFAS exhibit distinctive toxicokinetic characteristics-absorption, distribution, metabolism, and excretion (ADME) (Figure 1). These processes are driven not only by the extreme stability of the C-F bond but also by their unique amphiphilic nature (possessing both hydrophobic fluorinated tails and hydrophilic functional groups). These combined properties make PFAS highly susceptible to rapid absorption, extensive protein binding, selective tissue distribution, and resistance to biotransformation, ultimately resulting in long biological half-lives and high bioaccumulation potential in humans.

Humans can be exposed to PFAS through multiple pathways, primarily oral ingestion, inhalation, and dermal contact [18]. For the general population, ingestion is the dominant exposure route [19]. Oral ingestion of contaminated food and drinking water is the dominant exposure route, typically accounting for 70% to over 90% of total exposure [20]. In occupational settings, inhalation is the most common route of exposure, with possible contributions from dermal absorption and dust ingestion [21]. PFAS present in air, especially volatile or semi-volatile precursors, as well as PFAS bound to particulate matter, can enter the body via the respiratory system. Given that humans typically spend about 90% of their time indoors, and that indoor environments contain multiple PFAS emission sources, PFAS concentrations in indoor air and dust may be higher than outdoors, constituting a significant source of inhalation exposure [22]. A systematic review indicates that airborne PFAS levels are generally lowest outdoors (typically 0.5–3.0 ng/m^3^), significantly higher in general indoor residential settings (3.0–15.0 ng/m^3^), and can reach the highest levels, sometimes exceeding 100 ng/m^3^, in indoor occupational environments [23]. Similarly, PFAS concentrations in indoor dust, a primary sink for these chemicals, vary dramatically by microenvironment. Recent monitoring in Australia has revealed that offices exhibit significantly higher total PFAS concentrations in dust (mean ± SD: 400 ± 810 ng/g) compared to residential houses (170 ± 350 ng/g) and public transport vehicles (39 ± 33 ng/g), with PFOA frequently dominating the indoor profiles [24]. Furthermore, indoor fine particulate matter (PM_2.5_) has been shown to carry a wider diversity and higher concentration of ionic PFAS (e.g., perfluoroalkyl carboxylic acids, PFCAs) compared to outdoor ambient air. These PFAS are emitted from indoor materials and then partition onto inhalable PM_2.5_, thereby creating a direct exposure pathway [25]. Dermal exposure represents a non-negligible route. Current research has identified transporters such as organic anion transporters (OATs) and organic anion transporting polypeptides (OATPs) in the skin, which have been confirmed to participate in PFAS transport in other tissues, suggesting potential molecular mechanisms for transdermal PFAS absorption [26].

Once absorbed, PFAS are distributed throughout the body via the bloodstream. In vivo, PFAS primarily bind to serum proteins, particularly serum albumin and fatty acid-binding proteins (FABPs) [27], which facilitate their transport and distribution. PFAS accumulate mainly in protein-rich compartments such as blood, liver, and kidneys, rather than adipose tissue, and they are also capable of crossing the blood–brain barrier to reach the central nervous system [28]. Both phospholipids and proteins play important roles in the tissue distribution and accumulation of PFAS [29].

The metabolism of PFAS is complex and varies with carbon chain length. Short-chain PFAS (e.g., Perfluorobutanoic acid (PFBA) and Perfluorobutane sulfonate (PFBS)) are readily metabolized and excreted quickly from the body. In contrast, longer-chain PFAS (e.g., Perfluorohexane sulfonate (PFHxS), perfluorooctanoic sulfonate (PFOS), and Perfluorooctanoic acid (PFOA)) are metabolized more slowly. They have longer half-lives and are not easily metabolized [30].

Urinary excretion is the primary route for eliminating PFAS from the human body. Renal clearance of PFAS comprises three processes: glomerular filtration, tubular secretion, and tubular reabsorption [31]. The slow elimination of PFAS is attributable primarily to active reabsorption in the renal tubules, which decreases their clearance [32]. A small proportion of PFAS can also be excreted via feces, primarily associated with unabsorbed fractions or biliary excretion involving enterohepatic circulation [27]. Following biliary excretion into the intestine, some PFAS are reabsorbed and returned to the liver via the portal circulation [33]. This recirculation mechanism further impedes the elimination of PFAS from the body. PFAS can also be excreted via breast milk, which constitutes a maternal elimination route but a source of exposure for infants [26].

Overall, the ADME properties of PFAS, including high absorption rates, serum protein binding and limited excretion due to active renal reabsorption and enterohepatic circulation, determine their long-term accumulation in the human body. This persistent internal exposure enables prolonged interactions with biological systems and increases the likelihood of toxic effects across multiple organs. Accordingly, the following sections focus on the toxicity mechanisms and health impacts resulting from the accumulation of PFAS in humans.

## 3. Toxicity Mechanisms and Health Impacts

An increasing body of toxicological evidence indicates that PFAS exert diverse toxic effects by perturbing lipid and amino acid metabolism, disrupting multiple cellular signaling pathways, and binding to nuclear receptors [34]. The toxicity of PFAS does not arise from a single mechanism but involves multiple molecular and cellular pathways, the disturbances of which may ultimately lead to different adverse health outcomes in humans. Epidemiological findings further support some of these adverse effects.

### 3.1. Endocrine Disruption

**Figure 2:**
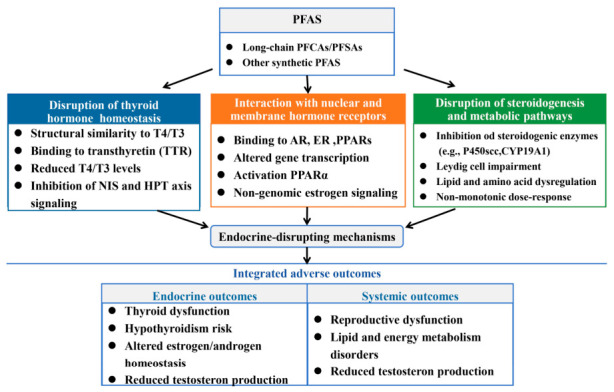
Endocrine-disrupting Mechanisms and Effects of PFAS.

PFAS exhibit typical endocrine-disrupting properties. They can interfere with the normal function of the endocrine system through multiple mechanisms, leading to adverse health effects (Figure 2). Consequently, some PFAS have been classified as endocrine-disrupting chemicals (EDCs) [35]. Extensive evidence suggests that endocrine disruption is a key mechanism underlying PFAS toxicity. By perturbing lipid and amino acid metabolism, disrupting cellular signaling pathways, and binding to nuclear receptors [34], PFAS adversely affect thyroid function, steroid hormone homeostasis, and metabolic regulation [36,37]. First, PFAS can disrupt thyroid hormone homeostasis, which may lead to altered metabolic regulation and developmental outcomes. Mechanistically, PFAS interfere with thyroid hormone synthesis, transport, and metabolism, and can disturb the hypothalamic-pituitary-thyroid axis as well as thyroid hormone receptor signaling [38]. Second, PFAS can disturb lipid and energy metabolism, partly by activating nuclear receptors such as peroxisome proliferator-activated receptors (PPARs) and altering key metabolic pathways involved in fatty acid metabolism and endocrine regulation [39]. Third, PFAS can alter steroid hormone balance, including estrogen and androgen signaling, mainly through direct interactions with nuclear hormone receptors and competitive binding that modifies receptor activity [40]. Together, these mechanisms illustrate how PFAS act as endocrine-disrupting chemicals affecting multiple hormonal systems and metabolic processes.

Disruption of thyroid hormone homeostasis is a significant health concern associated with PFAS exposure. The underlying mechanisms involve multiple steps in the synthesis, secretion, and transport of thyroid hormones (THs) [41]. PFAS, particularly PFOA and PFOS, exhibit certain structural similarities with THs, such as thyroxine (T4) and triiodothyronine (T3) [19]. This structural similarity enables PFAS to competitively bind to plasma transport proteins of thyroid hormones, primarily transthyretin (TTR). PFAS competes with T4 for binding to TTR, which may lead to decreased thyroid hormone levels and adverse endocrine-disrupting effects [42]. The longer the carbon chain of PFAS, the stronger the interaction with TTR [36]. This competitive binding results in a temporary rise in the concentration of free T4 in the blood and suppresses the hypothalamic-pituitary-thyroid (HPT) axis via negative feedback mechanisms. For instance, based on quantitative in vitro to in vivo extrapolation (QIVIVE) models, this displacement is predicted to lead to a median increase in free T4 concentrations of 2.57% (with a 95% prediction interval of 0.432–23.5%) under high occupational exposure to PFAS [43]. This ultimately leads to lower levels of circulating total thyroid hormone [41]. Several studies have found a negative correlation between exposure to PFAS and serum total T4 levels, which could potentially induce hypothyroidism [44]. Furthermore, PFAS can disrupt thyroid hormone synthesis by inhibiting the activity of the sodium iodide symporter (NIS), a transmembrane transport protein responsible for iodide uptake into thyroid follicular cells, thereby reducing intracellular iodine availability and limiting the substrate required for thyroid hormone production [19]. Additionally, molecular studies reveal that mid- to long-chain PFAS impact thyroid hormone synthesis by changing the local hydrogen bond network and the required orientation of hormonogenic residues within the key synthetic protein, human thyroglobulin (hTG), with the toxic effects of sulfonic PFAS being more prominent than those of carboxylic PFAS [45]. Consistent findings from epidemiological studies indicate that exposure to PFAS is associated with an increased risk of thyroid dysfunction in human populations [46]. Additionally, mechanistic studies suggest that PFAS may indirectly affect the thyroid system by influencing neural signaling in the hypothalamus and metabolic pathways in the liver, rather than acting directly on the pituitary or thyroid gland itself [34].

Nuclear hormone receptors (NHRs) are key regulators of endocrine signaling. In silico molecular-docking studies indicate that multiple PFAS, particularly long-chain PFCAs and perfluoroalkane sulfonic acids (PFSAs), exhibit binding potential to several NHRs, including the androgen receptor (AR), estrogen receptor (ER), and PPARs [34,36]. In vitro experiments further confirm the receptor-mediated effects of PFAS. Three manufactured PFASs namely (9-(nonafluorobutyl)-2,3,6,7-tetrahydro-1 H,5 H,11 H-pyrano[2,3-f]pyrido[3,2,1-ij]quinolin-11-one (NON), 2-(heptafluoropropyl)-3-phenylquinoxaline (HEP), and 2,2,3,3,4,4,5,5,5-nonafluoro-N-(4-nitrophenyl)pentanamide (NNN, have been proven in vitro to inhibit testosterone-induced transcriptional activation of the AR through a competitive binding mechanism and downregulate the expression of AR-responsive genes (such as PSA and FKBP5) [47]. In addition to classical nuclear receptor pathways, PFAS may also act through non-genomic estrogen signaling mechanisms. Recent studies suggest that PFOA can activate the G protein-coupled estrogen receptor (GPER), a membrane-associated estrogen receptor that mediates rapid intracellular signaling events. Activation of GPER by PFAS has therefore been proposed as a molecular initiating event (MIE) contributing to endocrine-disrupting effects through indirect estrogen-like signaling pathways [34].

Beyond acting on receptors, PFAS can directly interfere with the process of steroid hormone biosynthesis [34]. PFAS can disrupt the homeostasis of steroid hormones by inhibiting their synthesis and interfering with receptor-mediated processes. They may affect key enzymes in the steroid hormone synthesis pathway, such as the cholesterol side-chain cleavage enzyme (P450scc) and aromatase (CYP19A1), thereby interfering with the production of these hormones [46]. In vitro experiments have demonstrated that both PFOS and PFOA reduce the expression or activity of all steroidogenic enzymes [36]. However, some studies have suggested that low doses of PFOA may stimulate the synthesis of steroid hormones by accelerating steroidogenesis and fatty acid metabolism [34]. This indicates that the effects of PFAS may exhibit a non-monotonic dose–response relationship.

### 3.2. Immunotoxicity

Epidemiological studies have linked PFAS exposure to various immune-related diseases, such as asthma, allergies, and inflammatory bowel disease [48]. Authoritative agencies such as the European Food Safety Authority (EFSA) and the U.S. Environmental Protection Agency (EPA) have identified immunotoxicity, particularly the suppression of vaccine antibody responses, as a critical health effect in PFAS risk assessments [48,49]. According to a human biomonitoring study, the sum of serum concentrations of four major PFAS (PFOA, PFOS, PFHxS, and PFNA), ranging from 16.1 to 43.5 ng/mL, is classified as posing a moderate to severe risk for immunotoxicity [50]. However, the evidence regarding this association is not entirely consistent. A recent systematic review and meta-analysis indicated that, while there is evidence of a correlation between PFAS exposure and increased risk of infections in children, the evidence for a reduction in antibody titers is rated as ‘moderate to none’ [51]. This suggests that the impact of PFAS on the immune system is complex and multifaceted. An earlier review also noted that, due to inconsistencies in the methodologies and results of existing studies, and because the doses required to observe immunologic effects in animal experiments are much higher than the exposure levels in the general population, the use of immune modulation as a key endpoint in PFAS risk assessments should be approached with cautious [51].

Recent studies have revealed several potential cellular and molecular mechanisms underlying the immunotoxicity of PFAS. At the cellular level, population studies have found that PFAS exposure is associated with changes in various immune cell subpopulations in peripheral blood, including natural killer (NK) cells, T helper (Th) cells, and cytotoxic T (Tc) cells [52]. In vitro studies utilizing human T cells revealed that PFAS mixtures, even at environmentally relevant low concentrations of 2 ng/mL, significantly reduce the expression of activation markers (e.g., CD71) on CD4^+^ T cells and suppress the production of pro-inflammatory cytokines like IFN-γ and TNF-α in CD8^+^ and MAIT cells [53]. Furthermore, structure-activity relationship studies in THP-1-derived macrophages have established specific toxicity thresholds: the 24 h half-maximal toxic concentration (TC_50_) for PFOS is 63.0 ± 9.2 μM, while emerging alternatives, such as 6:2 FTOH, exhibit even higher potency with a TC_50_ of 6.3 ± 1.9 μM [54]. Further confirmation came from animal experiments and population validation, which showed that exposure to PFAS mixtures can alter hematological parameters (such as white blood cells, platelets, and hemoglobin) and directly cause ultrastructural damage to splenic lymphocytes, including cellular edema and chromatin condensation [55]. Specifically, in vivo models have demonstrated that exposure to PFOA and PFOS can cause a 15% and 13% decrease in antigen-specific antibody production in mice, respectively. Additionally, developmental immune models show that zebrafish larvae are more susceptible to alternative PFAS, exhibiting a 96 h LC_50_ of 2.4 mg/L for F-53B, coupled with dysregulated immune-related gene expression [56]. PFAS not only alter the function of immune cells but can also directly damage their structure and survival. The inhibitory effects of PFAS are also evident in innate immune cells. Studies have found that PFAS mixtures attenuate the adaptive immune response of basophils mediated by Immunoglobulin E (IgE) receptors, while leaving their innate immune response induced by N-Formylmethionyl-leucyl-phenylalanine (fMLP) unaffected [53].

At the molecular level, studies have revealed specific pathways through which PFAS affects immune function. Janssen et al. [57] discovered that several PFAS compounds, including PFOA, PFOS, perfluorononanoic acid (PFNA), and PFHxS, could significantly reduce the expression of recombination activation genes (RAG1 and RAG2) in human B lymphocytes. These genes are crucial for generating antibody diversity, so the suppression of their expression may directly impair the body’s ability to produce effective antibodies. This provides molecular evidence for the weakened vaccine response. Other proposed mechanisms include modulation of nuclear receptors such as PPARs, interference with key signaling pathways such as Nuclear factor kappa-light-chain-enhancer of activated B cells (NF-κB), disruption of calcium homeostasis in immune cells, induction of oxidative stress, and perturbation of fatty acid metabolism [49]. Toxicogenomic analyses have also confirmed a close association between PFAS exposure and the dysregulation of multiple immune- and inflammation-related pathways, including Th17 cell differentiation and the Janus kinase-Signal transducer and activator of transcription (JAK-STAT) signaling pathway [55]. Meanwhile, PFAS mixtures have been shown to downregulate the gene expression of chemokine receptors and Th1-type cytokines (e.g., IFNG) in MAIT cells, providing molecular support for their immunosuppressive effects [53].

Notably, there exists a susceptible window of PFAS exposure, particularly during the development of the immune system. Early-life exposure may lead to more significant and lasting adverse effects [49]. In summary, the available evidence suggests that PFAS can impair the normal functioning of the immune system via various pathways. Immunotoxicity is a key factor that must be considered in the health risk assessment of these compounds.

### 3.3. Hepatotoxicity

**Figure 3:**
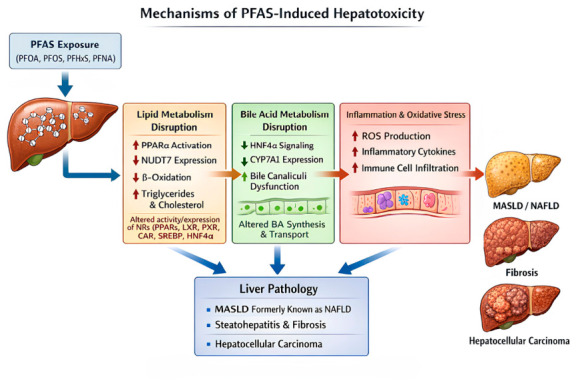
Mechanisms of PFAS-induced Hepatotoxicity.

The liver is a major target organ for PFAS toxicity because many PFAS accumulate preferentially in hepatic tissue and interact with key metabolic regulatory pathways. Evidence from in vitro cell models and in vivo animal studies indicates that PFAS can activate nuclear receptors, particularly PPARs, thereby altering hepatic lipid metabolism and bile acid homeostasis [58]. These disruptions contribute to metabolic dysfunction-associated steatotic liver disease (MASLD), formerly known as non-alcoholic fatty liver disease (NAFLD) [59], and may increase the risk of hepatocellular carcinoma [60]. Toxicity mechanisms are illustrated in Figure 3.

Disturbance of lipid metabolism is one of the most consistently reported hepatic effects of PFAS exposure. Epidemiological studies have shown that PFAS exposure is associated with elevated circulating lipid levels. For example, higher plasma concentrations of PFOS and PFHxS have been associated with increased levels of total cholesterol and lipid components across several lipoprotein subclasses, including HDL, IDL, LDL, and VLDL [61]. These metabolic alterations are considered important contributors to MASLD development.

Recent mechanistic studies have identified novel molecular pathways linking PFAS exposure to hepatic lipid accumulation. Gou et al. [62] reported that PFOA and PFOS suppress transcription of the Nudix hydrolase 7 (NUDT7) gene. NUDT7 encodes an acetyl-CoA hydrolase that regulates peroxisomal fatty acid β-oxidation. Downregulation of NUDT7 reduces fatty acid oxidation and promotes intracellular accumulation of triglycerides and cholesterol. Importantly, decreased NUDT7 expression has also been observed in the livers of patients with MASLD, suggesting a potential mechanistic link between PFAS exposure and human metabolic liver disease.

In addition to the NUDT7 pathway, various nuclear receptors are considered to be key molecular targets for the disruption of lipid metabolism induced by PFAS. PPARα is an important target for several PFAS, including PFOA and PFOS. Upon activation by PFOA or PFOS, PPARα can increase the expression of its downstream target gene, ACOX1, a key enzyme in peroxisomal β-oxidation. This process can lead to oxidative stress, impaired mitochondrial function, and lipid accumulation [63]. Structural biology studies further demonstrate that the molecular basis for PFOA-mediated activation of PPARγ, showing that PFOA acts as a partial agonist by binding to three distinct sites within its ligand-binding domain [64]. Other nuclear receptors also contribute to PFAS-induced metabolic dysregulation. Liver X receptor (LXR) regulate cholesterol transport and lipogenesis, while pregnane X receptor (PXR) and constitutive androstane receptor (CAR) regulate xenobiotic metabolism and lipid homeostasis [63]. Additionally, sterol regulatory element-binding proteins (SREBPs) control fatty acid synthesis [65,66], and hepatocyte nuclear factor-4α (HNF4α) regulates multiple genes involved in hepatic lipid metabolism [67]. PFAS exposure can alter the activity or expression of these transcription factors, thereby promoting hepatic lipid accumulation, triglyceride deposition, and progressive metabolic dysfunction [66,67,68].

Disruption of bile acid (BA) metabolism represents another important mechanism of PFAS-induced hepatotoxicity. PFAS exposure has been shown to alter bile acid synthesis and transport, potentially leading to cholestasis and further liver injury [69,70]. At the cellular level, exposure to PFOA and PFOS can cause dilation of bile canaliculi and redistribution of tight-junction proteins, which are morphological features consistent with cholestatic liver injury [71,72]. In vivo studies have also reported cholestasis and bile pigment deposition in the livers of mice exposed to PFOA or GenX [73]. At the molecular level, PFAS can disrupt bile acid homeostasis by altering the expression of key regulatory genes involved in BA synthesis and transport. For example, PFAS exposure can suppress HNF4α signaling and downregulate cholesterol 7-alpha-hydroxylase (Cyp7A1), the rate-limiting enzyme in bile acid synthesis [72]. However, regulation of Cyp7A1 appears to be compound-specific. Long-chain PFAS such as PFOA have been shown to suppress Cyp7A1 expression through activation of hepatic PPARα, whereas some short-chain PFAS (e.g., PFBA) may increase Cyp7A1 expression by inhibiting the intestinal FXR-FGF15 signaling pathway [74]. These differences indicate that distinct PFAS compounds can exert divergent regulatory effects on bile acid metabolism.

The liver, as the principal detoxification organ, mobilizes its metabolic and transport systems to respond to xenobiotic exposure, including PFAS [75]. Upon PFAS challenge, the hepatic detoxification system initiates an adaptive defense strategy, though this process is significantly compromised by both the direct and indirect effects of these chemicals. A direct mechanism of PFAS hepatotoxicity involves the interference with hepatic transporter function. For instance, legacy PFAS such as PFOA and PFOS act as direct inhibitors of key uptake transporters on the basolateral membrane of hepatocytes, including the Na+/taurocholate cotransporting polypeptide (NTCP) and (OATPs) [76]. Since these transporters are essential for the hepatic uptake and subsequent clearance of both endogenous compounds (e.g., bile acids) and exogenous toxins, their inhibition by PFAS can disrupt overall detoxification capacity. Indirectly, PFAS can act as activators of nuclear receptors. Several PFAS, including PFOA and PFOS, interact with and activate the CAR and PXR [77]. The persistent activation of these receptors, coupled with the environmental persistence of PFAS that prevents their effective clearance, leads to sustained dysregulation of genes at the intersection of xenobiotic metabolism and energy homeostasis [77]. This contributes to hepatic metabolic imbalance and promotes oxidative stress and mitochondrial dysfunction, which are key mechanisms in the exacerbation of liver injury [78].

Chronic liver injury induced by dysregulated lipid metabolism and disrupted bile acid homeostasis is often accompanied by inflammation and oxidative stress, and may ultimately progress to liver fibrosis and liver cancer [79]. Animal experiments have shown that PFOS and PFNA can induce inflammatory cell infiltration and increase the levels of inflammatory factors [71]. Lipidomic analyses have also revealed that exposure to emerging PFAS causes significant alterations in inflammation- and oxidative stress-associated lipid signaling molecules in mouse livers [80]. Although experimental studies demonstrate that certain PFAS, including PFOA and PFOS, can induce hepatocarcinogenesis in animal models, epidemiological evidence for liver cancer risk in humans remains limited and inconsistent [60].

### 3.4. Reproductive Toxicity

A large body of epidemiological and toxicological studies have indicated that PFAS exposure is closely associated with dysfunction of the male and female reproductive systems [19,81,82]. The mechanisms of their toxicity are complex, primarily involving endocrine disruption, oxidative stress, cytotoxicity, and genotoxicity, among other aspects.

#### 3.4.1. Male Reproductive Toxicity

Several mechanisms for PFAS-induced male reproductive toxicity have been revealed by experimental studies. Exposure to PFAS can cause damage to the testes and epididymis, thereby impairing spermatogenesis and sperm quality [83]. A primary mechanism of PFAS-induced male reproductive toxicity is the disruption of steroidogenesis in Leydig cells in the testes. Legacy PFAS and emerging alternatives such as perfluoro-3,5,7,9-tetraoxadecanoic acid (PFO4DA) have been shown to reduce testosterone levels by inhibiting the expression of key steroidogenic genes, particularly steroidogenic acute regulatory protein (StAR) and Cytochrome P450 Family 11 Subfamily A Member 1 (CYP11A1) [37,83]. This disruption is mediated by PPARα signaling. For example, PFOA has been shown to bind to and activate PPARα signaling, which alters the expression of genes involved in steroidogenesis and lipid metabolism in Leydig cells, ultimately leading to impaired testosterone production and reduced Leydig cell function [30]. Testosterone deficiency further impairs Sertoli cell function and the integrity of the blood-testis barrier (BTB), leading to disruption of spermatogenesis and compromised male reproductive health [37].

Sun et al. [83] mentioned that PFAS have been detected in human semen and may exert direct toxic effects on sperm. These mechanisms include inducing oxidative stress and abnormal calcium ion (Ca^2+^) influx, which affect sperm function. PFAS alter the lipid composition and fluidity of the sperm membrane to impair sperm motility and the ability to fertilize. In vitro experiments have also confirmed that exposure to PFOA damages the ability of human sperm to penetrate artificial cervical mucus. Mutalifu et al. [37] found that exposure to the emerging PFAS (e.g., PFO4DA) led to a significant decrease in sperm count and quality in mice. Epidemiological studies have also confirmed that PFOA impairs human sperm motility [44]. Furthermore, recent studies suggest that PFAS may cause genetic damage to germ cells. Research in Caenorhabditis elegans indicated that PFOA might induce DNA double-strand breaks, while PFNA increased the mutation rate in germ cells by interfering with the DNA mismatch repair pathway [84].

Although some findings are inconsistent, epidemiological studies also suggest that exposure to PFAS is adversely associated with several markers of male reproductive health. A systematic review of the epidemiological evidence by Petersen et al. [81] pointed out a lack of consistency across studies, which prevents drawing definitive conclusions at present. Data concerning the relationship between PFAS and male reproductive hormones, such as testosterone, luteinizing hormone (LH), and follicle-stimulating hormone (FSH), is complicated, with different studies reporting conflicting results. Some studies have found a negative correlation between serum PFOS levels and testosterone levels [83], while others have reported a positive association between PFOA levels and testosterone levels [85]. These experimental findings are strongly corroborated by recent epidemiological data. In a study of male adolescents residing near a fluorochemical facility (with a geometric mean serum total PFOS concentration of 8.4 μg/L), an interquartile range (IQR) increase in PFHxS was significantly associated with a 0.75-fold change in bioavailable testosterone and a 0.87-fold change in LH [86].

#### 3.4.2. Female Reproductive Toxicity

Exposure to PFAS is associated with various adverse outcomes affecting female reproductive health, including polycystic ovary syndrome (PCOS), premature ovarian insufficiency (POI), endometriosis, genital tract tumors, an increased risk of infertility, and abnormalities in the menstrual cycle [18,19,46,83]. However, research on the effects of PFAS on females is less comprehensive than research on males [46]. The primary toxic mechanism involves interference with the normal function of the hypothalamic–pituitary–ovarian (HPO) axis, disrupting the synthesis and signaling of sex hormones. PFAS may disrupt female reproductive function by inhibiting the critical kisspeptin signaling pathway in the hypothalamus. This inhibition reduces the secretion of gonadotropin-releasing hormone (GnRH) and LH, thereby disrupting the ovulatory cycle and potentially leading to ovulation failure [82]. Furthermore, PFAS can directly affect ovarian function through two major mechanisms. First, PFAS can disrupt ovarian steroidogenesis, thereby impairing follicular development. Experimental studies have shown that PFAS exposure can interfere with cholesterol transport and steroid hormone biosynthesis in ovarian cells, for example by inhibiting the StAR and altering the expression or activity of key steroidogenic enzymes. These changes reduce the synthesis of estradiol and progesterone, which are essential for normal follicle maturation and oocyte development [19,82]. Consistently, PFAS have been detected in follicular fluid, indicating that these compounds can directly alter the ovarian microenvironment and potentially impair follicle growth and oocyte maturation [18,46]. Second, PFAS may exert direct cytotoxic effects on ovarian cells. Experimental evidence suggests that PFAS exposure can induce oxidative stress and mitochondrial dysfunction in oocytes and granulosa cells, disrupt gap junctional intercellular communication (GJIC) between ovarian cells, and activate PPAR signaling pathways that alter lipid metabolism in ovarian tissue. These processes can interfere with oocyte meiosis, promote lipid accumulation in the ovary, and ultimately reduce oocyte viability and reproductive potential [18,82,85].

Despite increasing evidence linking PFAS exposure to female reproductive disorders, epidemiological findings remain inconsistent. For example, although several studies have reported positive associations between PFAS exposure (e.g., PFOA, PFOS, PFNA, and PFBS) and an increased risk of endometriosis [46,82,85], other studies have not observed significant associations [18,19]. These discrepancies may be related to differences in exposure levels, study populations, and the complex mixture nature of PFAS.

PFAS are chemicals capable of affecting the reproductive health of both genders. Their mechanisms of action are complex, involving multi-level interference with the endocrine system, from regulation of the HPO axis and hormone synthesis in target organs to cellular signaling and genetic damage. Despite extensive epidemiological research into the reproductive toxicity of PFAS, some findings remain inconsistent, and the underlying mechanisms are not fully understood. This highlights the necessity for further research.

### 3.5. Carcinogenicity

Regarding the carcinogenicity of PFAS, the International Agency for Research on Cancer (IARC) Working Group classified PFOA as carcinogenic to humans (Group 1) and PFOS as possibly carcinogenic to humans (Group 2B). PFOA was classified as ‘carcinogenic to humans’ (Group 1) based on ‘sufficient’ evidence of cancer in experimental animals and ‘strong’ mechanistic evidence in exposed humans, while PFOS was classified as ‘possibly carcinogenic to humans’ (Group 2B) based on ‘limited’ evidence of cancer in experimental animals and ‘inadequate’ evidence regarding cancer in humans [21]. The strength of epidemiological evidence varies by cancer type. The association between PFAS exposure (particularly PFOA) and kidney and testicular cancers is relatively consistent and sufficient. High levels of PFAS exposure significantly increase the risk of developing kidney and testicular cancers [87]. High levels of PFAS exposure increase the risk of developing kidney and testicular cancers [88]. For liver cancer, rodent studies have indicated that PFOA can induce liver tumors [87], and other studies have shown a potential link between PFOS levels and hepatocellular carcinoma (HCC) [89]. However, studies in humans have yielded inconsistent results, with some failing to find a clear association [88]. The relationship with breast cancer is even more complex. While some studies have reported a positive correlation between specific PFAS (such as PFOA and PFOS) and breast cancer risk [8], a comprehensive systematic review and meta-analysis concluded that the current evidence is insufficient to establish a definitive link due to significant heterogeneity among studies and differences in exposure assessment timepoints [90]. Furthermore, epidemiological studies have found that firefighters have a higher incidence of various cancers, including thyroid, kidney, testicular, and prostate cancers [91]. Therefore, research on the toxicity of PFAS should consider the cancer risk associated with occupational PFAS exposure.

The carcinogenic mechanisms of PFAS are considered to be multi-pathway and non-direct genotoxic, meaning they do not usually cause cancer directly by damaging DNA sequences [92]. The core mechanisms include endocrine disruption, metabolic reprogramming, epigenetic alterations, oxidative stress, chronic inflammation, and immunosuppression [8]. Due to their chemical structural similarity to fatty acids, PFAS can disrupt lipid and glucose metabolism by activating PPARs, particularly PPARα, and metabolic disorders are a key characteristic of cancer [92]. Another important mechanism is endocrine disruption. PFAS influence the development and progression of hormone-dependent cancers, such as prostate and breast cancer, by disrupting hormonal balance [8,92]. Additionally, PFAS affect gene expression by altering epigenetic modifications such as DNA methylation and create a favorable microenvironment for tumor initiation by activating chronic inflammatory responses and suppressing immune system functions (e.g., inhibiting cytokine secretion) [8]. In vitro studies have confirmed that various PFAS induce the production of reactive oxygen species (ROS), which may lead to oxidative DNA damage [3,93].

The carcinogenicity of PFAS is a complex process. Although current research provides evidence of an association between specific cancers and PFAS exposure, further confirmation is required through additional studies. Their carcinogenic mechanisms are not driven by a single pathway but rather by the synergistic effects of multiple interconnected biological processes, including oxidative stress, metabolic disorders, endocrine disruption, and epigenetic changes.

### 3.6. Other Health Impacts—Cardiovascular Disease

The association between PFAS and cardiovascular disease (CVD) has been investigated, but the evidence is contradictory and difficult to interpret. Epidemiological evidence linking PFAS exposure and clinical cardiovascular events such as myocardial infarction and stroke remain inconsistent [94]. These inconsistencies in the literature can be attributed to several methodological and conceptual factors. A primary factor is the predominance of cross-sectional study designs, which are highly susceptible to reverse causality and residual confounding [95,96]. And the temporal shift in PFAS exposure profiles contributes to conflicting results. With the phase-out of legacy long-chain PFAS, contemporary human exposure levels have decreased. Emerging evidence from populations with currently lower exposure levels suggests that some specific PFAS congeners (including certain legacy compounds) may exhibit paradoxical inverse associations with CVD risk, as observed in recent studies [95,97]. Moreover, the heterogeneity of PFAS compounds, such as differences between branched and linear isomers or short-chain replacements, means that aggregating total PFAS concentrations or relying on single time-point serum measurements can obscure specific toxicological effects, leading to mixed outcomes across different populations [96,97]. De Toni et al. [98] found an association between PFAS exposure and an increased risk of CVD. Epidemiological studies have linked PFAS exposure to risk factors for CVD, particularly dyslipidemia [99]. Several studies have consistently shown that exposure to different PFAS is associated with higher levels of total cholesterol and low-density lipoprotein cholesterol (LDL-C). However, it remains uncertain whether these biochemical changes directly correlate with an increased risk of cardiovascular events [100].

More recently, Wei et al. [101] reported that exposure to multiple PFAS, including PFOA, PFOS, PFHxS, and PFNA, was significantly associated with an increased risk of atherosclerotic cardiovascular disease (ASCVD). Similarly, a study of postmenopausal women identified PFOS as a strong predictor of coronary artery disease (CAD) and PFOA as a moderate predictor of coronary microvascular disease (CMD), potentially involving alterations in inflammatory signaling and amino-acid metabolic pathways [102]. In contrast, two large prospective cohort studies and a meta-analysis conducted in Sweden did not find significant associations between PFAS exposure and cardiovascular events such as myocardial infarction or stroke, and even reported a modest negative association between PFOA exposure and CVD risk [94,100].

The biological mechanisms linking PFAS exposure to cardiovascular disease are complex and likely involve multiple interacting pathways. One important mechanism involves metabolic disturbances. PFAS exposure has been associated with dyslipidemia, glucose metabolism disorders, and hypertension, which are well-established risk factors for atherosclerosis and CVD. These metabolic alterations may contribute to lipid accumulation in vascular tissues and accelerate atherosclerotic plaque formation [99,103].

Another key mechanism involves chronic inflammation and endothelial dysfunction. PFAS exposure has been associated with increased circulating levels of inflammatory markers, including interleukin-6 (IL-6) and tumor necrosis factor-α (TNF-α), which can promote vascular inflammation and contribute to the development of atherosclerosis [104]. Inflammatory activation may also impair endothelial function, further facilitating vascular injury and plaque development.

Kidney function may also mediate the cardiovascular effects of PFAS exposure. PFAS accumulate in the kidneys and can impair renal function, which is closely linked to cardiovascular health [105]. Declining kidney function may contribute to increased cardiovascular risk through mechanisms such as altered lipid metabolism, increased systemic inflammation, and impaired blood pressure regulation. Causal mediation analyses have shown that reduced kidney function may account for more than 33% of the association between PFAS exposure and ASCVD risk [101,105].

Platelet activation and thrombosis may represent another pathway linking PFAS exposure to cardiovascular disease. Experimental studies have shown that certain long-chain PFAS, such as PFTA and PFDA, can interact with the extracellular domain of platelet glycoprotein GPIbα, triggering intracellular calcium mobilization and activation of integrin αIIbβ3. These processes enhance platelet aggregation and thrombus formation, providing a potential mechanistic explanation for the increased cardiovascular risk associated with PFAS exposure [102].

## 4. Challenges and Future Research Directions

Research on the toxic effects of PFAS and their implications for human health is complicated by several factors. First, the PFAS class comprises thousands of structurally diverse compounds, and the substantial variability in toxicity profiles and modes of action across individual PFAS complicates cross-study comparisons and limits the generalizability of findings. Second, inconsistencies and limited standardization in current analytical methods for PFAS detection and quantification reduce the comparability of exposure measurements across studies. In addition, although epidemiological and experimental studies have reported associations between PFAS exposure and adverse health outcomes, the causal mechanisms and key biological targets underlying these effects remain incompletely characterized. Importantly, even low-level environmental exposure may be associated with measurable health risks, and toxic responses can vary by sex, species, and life stage. Existing evidence suggests that PFAS may impact human health through pathways related to endocrine disruption, immunotoxicity, hepatotoxicity, reproductive toxicity, carcinogenicity, and cardiovascular disease; however, the specific molecular and cellular mechanisms underlying these outcomes require further investigation. Therefore, future research should focus on the following aspects:

(1) Health impacts of low-dose, long-term exposure and mixtures

Future research should prioritize investigating the potential health effects of long-term, low-dose exposure on humans, particularly susceptible populations such as children, pregnant women, and the elderly. More attention should also be given to the combined toxic effects of PFAS mixtures. Developing novel experimental designs and data analysis methods is crucial to better simulate real-world exposure scenarios and clarify the toxic effects and health impacts of interactions between different PFAS compounds.

(2) In-depth exploration of toxicity mechanisms

Future studies should intensify efforts in PFAS toxicity research to fill existing data gaps and deeply investigate the toxicity mechanisms of different PFAS, especially their targets of action and signaling pathways at the cellular and molecular levels. It is essential to develop new research approaches that better simulate real-world exposure scenarios, clarify the molecular mechanisms of interaction, and determine the various dose–response relationships between PFAS and target endpoints. Expanding research on mechanisms of action, toxicokinetics, and adverse outcome pathways will help to clarify variations in responses across different species, sexes, and life stages.

(3) Interdisciplinary research and collaboration

Promoting interdisciplinary research that integrates environmental science, toxicology, epidemiology, and clinical medicine is vital. Through cross-disciplinary collaboration, a comprehensive assessment of the environmental behavior, exposure pathways, toxic effects, and health impacts of PFAS can be achieved. Establishing international collaborative research networks to share data and resources will improve research efficiency and quality. Furthermore, based on new research findings, PFAS health risk assessment models will help inform the development of more scientific and rational environmental standards and health guidance values.

## 5. Conclusions

Based on current research, PFAS have adverse effects on human health; however, their toxicological mechanisms are not yet fully elucidated. Given the inconsistencies among some studies and epidemiological findings, further in-depth investigation is required. In conclusion, addressing the health challenges posed by PFAS requires in-depth exploration and innovation in future research to better protect human health and environmental safety.

## Data Availability

No new data were created or analyzed in this study. Data sharing is not applicable to this article.

## Abbreviations

The following abbreviations are used in this manuscript:

PFAS	Per- and polyfluoroalkyl substances
C-F	Carbon-fluorine
ADME	Absorption, distribution, metabolism, and excretion
OATs	Organic anion transporters
OATPs	Organic anion transporting polypeptides
FABPs	Fatty acid-binding proteins
PFBA	Perfluorobutanoic acid
PFBS	Perfluorobutane sulfonate
PFHxS	Perfluorohexane sulfonate
PFOS	Perfluorooctanoic sulfonate
PFOA	Perfluorooctanoic acid
EDCs	Endocrine-disrupting chemicals
PPAR	Peroxisome proliferator-activated receptor
THs	Thyroid hormones
T4	Thyroxine
T3	Triiodothyronine
TTR	Transthyretin
HPT	Hypothalamic-pituitary-thyroid
NIS	Sodium iodide symporter
NHRs	Nuclear hormone receptors
PFCAs	Perfluoroalkyl carboxylic acids
PFSAs	Perfluoroalkane sulfonic acids
AR	Androgen receptor
GPER	G protein-coupled estrogen receptor
P450scc	Cholesterol side-chain cleavage enzyme
CYP19A1	Aromatase
EFSA	European Food Safety Authority
EPA	Environmental Protection Agency
NK	Natural killer cell
Th	T helper cell
Tc	Cytotoxic T cell
IgE	Immunoglobulin E
fMLP	N-Formylmethionyl-leucyl-phenylalanine
PFNA	Perfluorononanoic acid
RAG	Recombination activation gene
NF-κB	Nuclear factor kappa-light-chain-enhancer of activated B cells
JAK-STAT	Janus kinase-Signal transducer and activator of transcription
MAIT	Mucosal-associated invariant T cell
MASLD	Metabolic dysfunction-associated steatotic liver disease
NAFLD	Non-alcoholic fatty liver disease
NUDT7	Nudix hydrolase 7
LXR	Liver X receptor
PXR	Pregnane X receptor
CAR	Constitutive androstane receptor
SREBPs	Sterol regulatory element-binding proteins
HNF4α	Hepatocyte nuclear factor-4α
BA	Bile acid
Cyp7A1	Cholesterol 7-alpha-hydroxylase
PFO4DA	Perfluoro-3,5,7,9-tetraoxadodecanoic acid
StAR	Steroidogenic acute regulatory protein
CYP11A1	Cytochrome P450 Family 11 Subfamily A Member 1
BTB	Blood-testis barrier
LH	Luteinizing hormone
FSH	Follicle-stimulating hormone
PCOS	Polycystic ovary syndrome
POI	Premature ovarian insufficiency
HPO	Hypothalamic–pituitary–ovarian
GnRH	Gonadotropin-releasing hormone
GJIC	Gap junctional intercellular communication
IARC	International Agency for Research on Cancer
HCC	Hepatocellular carcinoma
ROS	Reactive oxygen species
CVD	Cardiovascular disease
LDL-C	Low-density lipoprotein cholesterol
ASCVD	Atherosclerotic cardiovascular disease
CAD	Coronary artery disease
CMD	Coronary microvascular disease
IL-6	Interleukin-6
TNF-α	Tumor necrosis factor-α
